# Effect of clinical pharmacists participating in nutritional therapy for patients with acute cerebral infarction complicated with dysphagia

**DOI:** 10.12669/pjms.39.4.7121

**Published:** 2023

**Authors:** Lixin Wu, Haiyan Hou

**Affiliations:** 1 Lixin Wu Department of Pharmacy, Baoding No.1 Central Hospital, Baoding 071000, Hebei, China; 2 Haiyan Hou Department of Gastrointestinal Surgery, Dingzhou People’s Hospital, Baoding 073000, Hebei, China

**Keywords:** Acute cerebral infarction complicated with dysphagia, Nutritional therapy, Pharmaceutical intervention, Nutritional status, Cerebral neurological function, Adverse event

## Abstract

**Objective:**

To explore the effect of clinical pharmacists participating in nutritional therapy for patients with acute cerebral infarction (ACI) complicated with dysphagia.

**Methods:**

This is a Clinical comparative study. A total of 82 patients with ACI complicated with dysphagia treated in Baoding No.1 Central Hospital from May 2021 to February 2022 were included as subjects. They were divided into control group (n= 40, without clinical pharmacists) and experimental group (n= 42, with clinical pharmacists) using a random number table. The effect of nutritional therapy and the incidence of adverse reactions were compared between the two groups.

**Results:**

In the experimental group, PALB and ALB were both higher than those in the control group on the seven and 14-day after treatment (*p<* 0.05), while HB was higher than that in the control group only on the 14-day after treatment (*p<* 0.05). After treatment for 14-day, MAMC and TSF in the experimental group were higher than those in the control group (*p<* 0.05), while NIHSS score was lower than that in the control group (*p<* 0.05). The incidence of adverse events in the experimental group was lower than that in the control group (*p<* 0.05).

**Conclusion:**

Pharmaceutical intervention in nutritional therapy for patients with ACI complicated with dysphagia has positive significance in further improving the nutritional status and nutritional indexes, enhancing the efficacy of drug treatment and reducing the risk of adverse events, and is worthy of promotion.

## INTRODUCTION

Acute cerebral infarction (ACI) is a common cerebrovascular disease, which is a symptom of tissue necrosis and softening caused by localized hypoxia or ischemia of the brain.^1^,^2^ Patients often have dysphagia due to impaired brain nerve function.^3^ It has been pointed out^4^ that patients with cerebral infarction accompanied by dysphagia usually have malnutrition to varying degrees. At present, nutritional support is often conducted in different ways in clinic to improve the body resistance and prognosis of patients.^5^ There are two nutritional support methods that can be used for patients with ACI accompanied by dysphagia: enteral nutrition (EN) and parenteral nutrition (PN).

A study^6^ has pointed out that compared with PN, EN is more in line with human physiological characteristics, with low body rejection and risk of complications, and it is the preferred regime at present. When some nutrients in the blood of the body are higher than their normal ranges, they can play a variety of pharmacological effects such as anti-inflammation, immune regulation and gastrointestinal barrier.^7^ Moreover, clinical pharmacists play an important role in the clinical treatment and rational drug use of various patients.^8^ On this basis, this study will explore the effect of pharmaceutical intervention on nutritional therapy of patients with ACI complicated with dysphagia.

## METHODS

This is a Clinical comparative study. A total of 82 patients with ACI complicated with dysphagia treated in Baoding No.1 Central Hospital from May 2021 to February 2022 were included as subjects. Using a random number table, the patients were divided into control group (n= 40) and experimental group (n= 42). No significant differences were found between the two groups in terms of gender, age, onset time and BMI (*p>* 0.05), suggesting comparability.

### Ethical Approval:

The study was approved by the Institutional Ethics Committee of Baoding No.1 Central Hospital (No.: [2021] 039; date: May 27, 2021), and written informed consent was obtained from all participants.

### Inclusion criteria:

- Patients meeting the diagnosis of acute ischemic stroke;^9^
- Patients were accompanied by dysphagia at different degrees;
- Patients receiving EN or PN;
- Patients or their family members signed the informed consent, and the study was approved by the Ethics Committee of our hospital.

### Exclusion criteria:

- Abnormal physiological structure of the upper digestive tract;
- Complicated with other gastrointestinal dysfunctions;
- Inconsistent with the indications for EN and PN;
- Severe malnutrition;
- Malignant tumors, abnormal hematopoiesis and other diseases;

No pharmacists were involved in the control group during treatment. According to the European Guidelines for Treatment of Acute Cerebral Infarction,^10^ routine clinical treatment was carried out for the patients. Routine cardiac monitoring, blood pressure management, targeted oxygen inhalation, and general treatment such as lipid regulation, blood pressure reduction, maintenance of water-electrolyte balance, blood glucose and body temperature control were given. Patients with onset time < six hours were admitted to the stroke unit timely and given intravenous thrombolytic therapy, cooperated with antiplatelet aggregation, anticoagulation and neuroprotective therapy. According to the gastrointestinal function of patients, corresponding nutritional therapy was performed. Patients with gastrointestinal dysfunction were given PN, and those without abnormalities were given EN.

In the experimental group, pharmacists participated in the treatment and provided corresponding pharmaceutical care based on the above treatment. Firstly, the clinical pharmacists made a comprehensive assessment of the overall nutritional status of the patients in this group in combination with the Nutrition Risk Screening 2002 (NRS2002). In combination with the results of nutrition assessment, a reasonable dietary plan was formulated by nursing staff. According to the nutritional status of patients, the dosage and course of nutrient infusion were reasonably planned. Pharmaceutical care was provided during the whole treatment. Close attention was paid to the changes in the nutritional status of the patients.

### Observation indicators:

Main evaluation indicators: The nutritional indexes and nutritional status of the two groups before and after treatment were used as the main evaluation indicators. (1) Peripheral venous blood (five ml) was collected from the patients as samples. Nutritional indexes such as hemoglobin (HB), prealbumin (PALB) and serum albumin (ALB) were detected and recorded. (2) The nutritional status of the two groups was observed with triceps skinfold thickness (TSF) and mid-arm muscle circumference (MAMC) of the non-paralyzed side as evaluation indicators. Normal value of TSF: ≥ 12.5 mm for males and ≥ 16.5 mm for females, and it is normal if it is <10% of the normal value. Normal value of MAMC: the midline circumference of the mid-arm was measured, and the calculation formula was: MAMC (mm) = midline circumference of the mid-arm (mm) - 3.14 × TSF (mm).

### Secondary evaluation indicators:

The cerebral neurological function and the incidence of adverse events of the two groups before and after treatment were used as secondary evaluation indicators. The neurological function was assessed comprehensively using the National Institutes of Health Stroke Scale (NIHSS). The scoring criteria of NIHSS:^11^ the theoretical score range of 0-42, the higher the NIHSS score, the severer the neurological deficit. In this study, the adverse events of the two groups after treatment mainly included pulmonary infection, gastrointestinal reaction, urinary system infection and blood glucose elevation. The sum of patients with the above symptoms was taken as the final incidence for comparison.

### Statistical Analysis:

All data were included in SPSS 26.0 for statistical analysis. The measurement data were expressed as mean ± standard deviation (*χ̅*±*S*), and compared using the independent-sample t-test. The enumeration data were expressed as cases (n) and percentages (%), and compared by the chi-square (χ^2^) test. P*<* 0.05 was considered statistically significant.

## RESULTS

In the experimental group, PALB and ALB were both higher than those in the control group on the seven and 14-day after treatment (*p<* 0.05), while HB was higher than that in the control group only on the 14-day after treatment (*p<* 0.05). On the seven and 14-day after treatment, PALB and ALB decreased in both groups compared with those on one d (*p<* 0.01), while HB only reduced in the control group compared with that on one d (*p<* 0.05) (Table-I). MAMC and TSF in the experimental group on the 14-day after treatment were both higher than those in the control group (*p<* 0.05).

**Table-I:** Comparison of nutritional indexes (*χ̅*±*S*).

Index	Time	Experimental group	Control group	t	p
HB	1 d	132.98 ± 23.19	138.53 ± 23.28	-1.081	0.283
	7 d	129.83 ± 17.68	124.07 ± 19.71*	1.396	0.167
	14 d	126.65 ± 16.67	117.48 ± 20.07**	2.256	0.027
PALB	1 d	230.92 ± 36.55	225.18 ± 42.92	0.652	0.516
	7 d	192.22 ± 55.28**	165.16 ± 50.53**	2.311	0.023
	14 d	199.03 ± 46.66**	169.69 ± 42.59**	2.969	0.004
ALB	1 d	38.87 ± 4.80	39.14 ± 5.43	-0.242	0.809
	7 d	34.20 ± 4.88**	31.17 ± 5.08**	2.747	0.007
	14 d	34.39 ± 5.24**	31.38 ± 3.27**	3.140	0.002

***Notes:*** Compared in the group on one day after treatment,

* p< 0.05,

** p< 0.01.

On the 14-day after treatment, MAMC and TSF decreased in both groups compared with those on the one-d (*p<* 0.05) (Table-II). After treatment for 14-day NIHSS score in both groups was lower than that on the one-d after treatment (*p<* 0.01), and NIHSS score in the experimental group was lower than that in the control group (*p<* 0.05) (Table-III). After treatment for 14-day the incidence of adverse events in the experimental group was 19.05%, which was lower than 42.50% in the control group (*p<* 0.05) (Table-IV).

**Table-II:** Comparison of nutritional status between two groups before and after treatment (*χ̅*±*S*).

Indicator	Time	Experimental group	Control group	t	p
MAMC	1 d	24.10 ± 0.33	24.05 ± 0.43	0.593	0.555
	14 d	23.85 ± 0.45**	23.48 ± 0.79**	2.616	0.011
TSF	1 d	13.91 ± 0.35	13.87 ± 0.40	0.563	0.575
	14 d	13.73 ± 0.39*	13.47 ± 0.46**	2.781	0.007

***Notes:*** Compared in the group on 1 d after treatment,

* p< 0.05,

** p< 0.01.

**Table-III:** Comparison of cerebral neurological function between two groups (*χ̅*±*S*, score).

Indicator	Time	Experimental group	Control group	t	p
NIHSS	1 d	14.62 ± 8.31	13.45 ± 4.69	0.790	0.432
	14 d	7.76 ± 5.24**	10.45 ± 4.76**	-2.43	0.017

***Notes:*** Compared in the group on 1 d after treatment, *p< 0.05,

** P<0.01

**Table-IV:** Comparison in incidence of adverse events between two groups (n, %).

Group	n	Pulmonary infection	Gastrointestinal reaction	Urinary system infection	Blood Glucose elevation	Incidence of adverse events
Experimental group	42	4	2	2	0	8 (19.05%)
Control group	40	11	4	1	1	17 (42.50%)
x^2^						5.317
P						0.021

## DISCUSSION

Our results showed that both groups achieved certain effects after nutritional therapy. Nutritional therapy is of great significance to maintain vital signs, improving prognosis and enhance survival rate of various critically ill patients.^12^ In this study, based on ASPEN clinical guidelines, EN or PN was given to the patients in the two groups correspondingly. After treatment, all nutritional indexes and neurological recovery were significantly improved compared with those before treatment. But in contrast, the therapeutic effect of the experimental group under pharmaceutical intervention was higher. PALB and ALB in the experimental group were both higher than those in the control group on the seven and 14 d after treatment, and HB was higher than that in the control group on the 14 days after treatment.

The reason is considered as follows: the clinical pharmacists participated in the whole nutritional therapy of the patients in the experimental group, and provided the individualized medication scheme in combination with the symptoms and signs of the patients. Pharmacists can clarify the indications and contraindications of various therapeutic drugs according to the patient’s situation, and the nutritional therapy formulated on this basis is more targeted and has higher efficacy.^13^

After treatment, although the MAMC and TSF of the two groups decreased compared with those before treatment, the MAMC and TSF of the experimental group were higher than those of the control group. And the results revealed that after pharmaceutical intervention, the NIHSS of the experimental group was lower than that of the control group, indicating that nutritional therapy under pharmaceutical intervention also has positive significance in improving the clinical symptoms of patients and improving the overall therapeutic efficacy.

ACI is characterized by acute onset and rapid condition changes. Clinically, such patients will be complicated by limb disorders or dysphagia on the affected side.^14^,^15^ Due to the decline in ingestion, the normal nutritional needs of the patients with cerebral infarction complicated with dysphagia cannot be met, thereby resulting in a high risk of malnutrition.^16^,^17^ Although the current nutritional therapy for such patients is still based on EN, the key to selecting a specific therapeutic way lies in whether the patient has gastrointestinal dysfunction.^18^

Clinical pharmacists have important role in guiding various patients to use drugs rationally. In recent years, with the continuous development of the theory of nutritional pharmacology, increasing number of researchers have recognized the pharmacological effects of various nutrients in nutritional therapy.^19^ To further optimize the nutritional therapy for patients with ACI, this study explored the effect of pharmaceutical intervention on nutritional therapy in such patients based on nutritional pharmacology.

A current study^20^ has shown that when the levels of specific nutrients in the blood increase, certain pharmacological mechanisms can be produced in the body. In the present study, the incidence of adverse events in the experimental group was significantly lower than the control group. The pharmacists in the experimental group firstly made a comprehensive assessment of the nutritional status of the patients in combination with nutrition assessment tools such as NRS2002.

### Limitations of the study:

It includes a small number of patients were included, with short follow-up time. In our future work, we will continue to enlarge the sample size and longer follow-up time, with the expectation of elaborating the long-term effect and benefit of this treatment scheme to patients in more detail.

## CONCLUSIONS

After formulating a reasonable nutrient ratio and a reasonable medication regimen, and carrying out whole pharmaceutical monitoring on the patients, the risk of adverse events during the treatment can be reduced to the greatest extent. Pharmaceutical intervention in nutritional therapy for patients with ACI complicated with dysphagia can significantly improve the nutritional status, further enhance the efficacy and reduce the risk of adverse events in the patients, so as to improve their prognosis.

### Authors’ Contributions:

**Lixin Wu and Haiyan Hou:** Designed this study and prepared this manuscript, and are responsible and accountable for the accuracy or integrity of the work.
